# Water Interactions in Hybrid Polyacrylate-Silicate Hydrogel Systems

**DOI:** 10.3390/ma13184092

**Published:** 2020-09-15

**Authors:** Joanna Mastalska-Popławska, Agata Stempkowska, Iwona Habina-Skrzyniarz, Artur T. Krzyżak, Paweł Rutkowski, Piotr Izak, Jakub Rudny, Tomasz Gawenda

**Affiliations:** 1 Faculty of Materials Science and Ceramics, AGH University of Science and Technology, Mickiewicza 30 Av., 30-059 Krakow, Poland; pawelr@agh.edu.pl (P.R.); izak@agh.edu.pl (P.I.); rudnyjakub@gmail.com (J.R.); 2 Faculty of Mining and Geoengineering, AGH University of Science and Technology, Mickiewicza 30 Av., 30-059 Krakow, Poland; stemp@agh.edu.pl (A.S.); gawenda@agh.edu.pl (T.G.); 3 Faculty of Geology, Geophysics and Environmental Protection, AGH University of Science and Technology, Mickiewicza 30 Av., 30-059 Krakow, Poland; ihabina@agh.edu.pl (I.H.-S.); akrzyzak@agh.edu.pl (A.T.K.)

**Keywords:** hybrid hydrogels, sodium water glass, porosity, relaxation time, viscoelasticity

## Abstract

Hybrid polyacrylate-silicate hydrogels were obtained in the presence of *N*,*N*′-methylenebisacrylamide (NNMBA) as the cross-linking monomer and sodium thiosulphate/potassium persulphate (NTS/KPS) as the redox initiators. The results of the tests allowed us to conclude that a hybrid structure with a polyacrylate scaffolding and a silicate matrix had been obtained. The results of the rheological analysis revealed that the hydrogel sample with a 1:7 mass ratio of sodium water glass to the sodium polyacrylate is characterized by the highest complex viscosity. Thermal analysis (Thermogravimetry/Differential Scanning Calorimetry (TG/DSC)) showed that water begins to evaporate at higher temperatures, from 120 °C to even 180 °C. These results were confirmed by mid-infrared spectroscopy (MIR) and nuclear magnetic resonance spectroscopy (NMR) analysis. Differences in the intensity of the peaks derived from water in the MIR spectra indicate that most of the water is bounded. In turn, NMR results showed that the mobility of water molecules decreases as the amount of sodium water glass in the mixture increases.

## 1. Introduction

A hydrogel is a type of system in which the dispersion phase is a polymer, while the dispersed phase is water. Water absorption, otherwise known as swelling, depends on, inter alia, the pH, ionic strength of the solution, temperature, and the degree of cross-linking of the polymer. Water absorption by hydrogels is possible due to the physical or chemical cross-linking of their structure, which also means that the hydrophilic polymer chains are not dissolved in the aqueous environment [1,2,3,4]. Chemical cross-linking may involve the introduction of the cross-linking monomer (e.g., NNMBA or ethylene glycol dimethacrylate (EGDMA)) into the system, reaction between complementary functional groups (e.g., OH^-^, COOH^-^, NH_2_^−^), delivery of the high-power radiation (e.g., gamma and electron beam) or the use of enzymes. This leads to the formation of the covalent bonds in the three-dimensional (3D) structure of the polymer network. However, physical cross-linking involves the creation of molecular entanglements in the 3D structure of the van der Waals type, ionic bonds, hydrogen bonds, microcrystals, etc. [1,2,5,6,7].

Hydrogels can absorb significant amounts of a solution, sometimes even several hundred times their initial mass. As a result, they have found applications in many areas of life such as cosmetics, pharmaceuticals, construction, gardening, and environmental protection. Water found in hydrogels can be divided into three types, i.e., free, intermediate and bound. The first is called freeze water and it can be compared to bulk water because its particles do not interact with the polymer chain and undergo thermal changes at 0 °C like bulk water. Intermediate water is also known as freezable bound water, which means that this type of water is poorly attached to the polymer chain and interacts weakly with bound water, as well as undergoing thermal changes at temperatures below 0 °C. Bound water does not freeze (that is why it is called non-freezing water) because it is closely related to the polymer chain and does not show first order changes in the temperature range from −70 to 0 °C [8,9,10,11,12,13,14,15,16,17,18,19,20,21].

**Figure 1:**
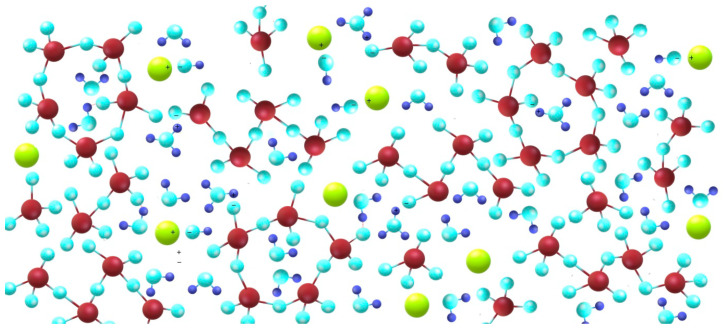
Schematic diagram of the water glass structures (dark red—silicon, light blue—oxygen, dark blue—hydrogen, green—sodium).

Aqueous soluble silicate solutions, known also as water glasses, are mixtures of cyclic and linear oligomers formed as the result of cracking of the silicon-oxygen bonds. The type of water glass is characterized by the silicate modulus, also known as the molar modulus (molar ratio of silica to the corresponding oxide), density, viscosity, and pH. The chemical structure of water glass is not known precisely. It can be considered as a solution, colloid or even a polymer. There are constant particle motions in water glass solutions. Hydration and ion exchange processes occurring between the phases determine the equilibrium state. The most suitable seems to be the mutual penetration of various structures, which can be confirmed by various physicochemical tests (e.g., thermal analysis (TG/DSC), IR, Raman and NMR spectroscopy) [22,23,24,25,26,27]. Silicate particles in an aqueous solution have a nucleus and are built of negatively charged structures whose electrostatic stabilization is ensured by alkali ions in such a way that some of these ions are inside the micelles, some are adsorbed on the surface, and other alkali ions, such as the ionised particles, are in the solution. It is recognized that the basic element of the alkali metal silicate structure is the [SiO_3_]^−2^ ion. This molecule easily attaches cations (in the case of solid silicates, they are usually metal cations). The phenomenon of cation attraction leads to the disruption of the silicate spatial network. Si–O bonds are broken and bonds of the alkali cation with oxygen are formed. During this process, ring and chain systems may be formed, as well as more [SiO_4_]^4−^ tetrahedrons. The pH of the solution also affects the structure of individual ions [28,29], influencing whether there are more or less charged ions in the system affecting the configuration of silicate units. At pH below 9, there are negatively charged colloidal SiO_2_ molecules. At the pH = 10.9, we have polyions. However, above this range, ions of [Si_2_O_5_]^2−^ type (pH = 10.9–13.6) and [SiO_3_]^2−^ type (pH > 13.6) predominate. Water in such systems can be considered to be similar to that in polymers. Negatively charged silicate structures interact with alkali metal cations (Na^+^, K^+^, Li^+^), as well as with the water molecules, building them into its micellar structure. Other water molecules are considered to be free water. Figure 1 shows a diagram of the water glass structures over a longer range.

Hybrid gels are most often polymer matrices containing various adjuvants in their structure, such as biological particles (peptides, proteins) or nano/microstructures, e.g., ceramic particles (titanium oxide etc.), that are physically or chemically connected to the polymer chains. They are most often used for biomedical purposes and their synthesis takes place in a neutral environment because of the high sensitivity of the used monomers to pH changes [1,8,30,31,32,33,34]. For example, in the case of acrylic monomers, the degree of polymerization is the highest in the pH range 7–10, while outside this range, at both low and high pH, there are phenomena interfering with the polymerization process [35,36,37,38]. Different types of water are present in this type of hybrid system and, due to their specific structure and polarity, they behave differently with ionic and non-ionic substances. The acceptor-donor properties of water also cause the formation of H_3_O^+^ and OH^−^ ions, and the hydrogen-oxygen bond (0.99 Å) in the molecule has a covalent polarized character. Water molecule has no linear shape, because the angle between the H–O–H bonds is about 105°, so the charge distribution is asymmetrical, causing the formation of dipoles. Orienting the dipoles with opposite poles causes the formation of fairly stable proton bridges, the breaking of which causes the additional dissociation of water. Summing up, thanks to the polarity and the possibility of forming of the hydrogen bonds, water has great potential for interaction.

There is very little information in the literature on the synthesis of silicate-polymer hydrogels obtained at high pH. Studies with various NMR methods on polymers or silica aqua solutions and gels have been conducted for a number of years [39,40,41]. Investigation of their mixtures was sporadically carried out [42]. In our earlier works [42,43], we presented the method of preparation and physicochemical characterization of hydrogels based on sodium acrylate and sodium water glasses. In this paper, we focus on presenting the mechanism of polymerization/gelation of the discussed silicate-polymer systems. We examine the impact of various types of water and its contribution on the physicochemical properties of these hydrogels and assess the impact of pH in this respect. Our approach also offers a complementary attitude enabling precise measurements of relaxation times by low-field nuclear magnetic resonance (LF-NMR) as well as diffusion coefficients using NMR Mobile Universal Surface Explorer (MoUSE) system. The information obtained will contribute to the development of the current state of knowledge about the types of water found in polymer and quazi-polymer (silicate) polar and non-polar structures, as well as answering the question of which medium, polymer or silicate, has a greater impact on the polymerization/gelation process of the silicate-polymer hydrogels. The results of our research can also be used in the designing of hydrogel hybrid materials for applications such as fire-retardant materials (use of a large water capacity of the hydrogel that can be controlled by the addition of water glass, which is also a well-known fire retardant) or for designing of the MRI phantoms for functional imaging, where, by the addition of paramagnetic ions such as sodium ions, we can control the relaxation phenomena.

## 2. Materials and Methods

### 2.1. Materials

Hydrogels were synthesized using the following reagents:

– Sodium water glass R-150 (WG; 29.1 wt.% of SiO_2_ and 14.70 wt.% of Na_2_O; silicate modulus M = 2.10; “Rudniki” Chemical Plant, Rudniki, Poland);
– 20 wt.% aqueous solution of sodium acrylate (ANa) synthesized in laboratory with the use of the saturated solution of sodium hydroxide (NaOH, Stanlab SJ, Lublin, Poland) and acrylic acid (AA; Acros Organics, Geel, Belgium);
– Potassium persulphate (KPS; POCh LLC, Geel, Poland);
– Sodium thiosulphate (NTS; POCh LLC, Gliwice, Poland);
– *N*,*N*′-methylenebisacrylamide (NNMBA; Acros Organics, Geel, Belgium).

### 2.2. Synthesis of Cross-Linked Polymer-Silicate Hydrogels

Polymer-silicate hydrogel samples were synthesized according to the scheme described in our previous publications [42,43]. Their compositions are presented in Table 1. For comparison, pure sodium water glass R-150 and pure sodium polyacrylate (pANa) were also tested. Due to the fact that the effect of water on the physicochemical properties of silicate-polymer hydrogels was examined, Table 1 also includes calculations regarding water content and information about the physical state of samples after polymerization.

### 2.3. Methods

Thermal analysis tests thermogravimetry/differential scanning calorimetry (TG/DSC) were carried out using a NETZSCH STA 449 F3 thermal analyzer (Selb, Germany) and aluminum oxide crucibles, in air atmosphere. Samples were heated up to 200 °C at a heating rate of 2 °C/min. TG was used to determine the weight loss of a polymer sample resulting from the release of free and bound water from the hydrogel structure. With the use of the DSC method, enthalpy changes (ΔH) of the tested samples occurring due to endothermic (−ΔH) or exothermic (+ΔH) transitions were determined.

An Excalibur BIO-RAD 6000 spectrometer (Hercules, CA, USA) equipped with an attenuated total reflectance (ATR) optical disk was used to identify water, as well as silicate and polymer functional groups in the analyzed silicate-polymer hydrogel samples. The spectra were recorded at a resolution of 1 and 25 scans in the range of 400–4000 cm^−1^.

NMR 1D T_1_ and T_2_ measurements were carried out on a Magritek Rock Core Analyzer (Wellington, New Zealand) equipped with a Halbach magnet with 0.05 T magnetic field using Inversion Recovery (IR) and Carr-Purcell-Meiboom-Gil (CPMG) sequences. IR experiments were obtained with 20 steps from 0.05 ms to 5 s. For CPMG50, 000 echoes with echo time (TE) of 100 µs were recorded. An inter-experiment delay of 5 s was used for both measurements. Data after background subtraction were inverted using Inverse Laplace Transform (ILT) with Lawson&Hanson algorithm within the Prospa software. NMR diffusion measurements were performed on a Mobile Universal Surface Explorer (MoUSE) system (Aachen, Germany) at a magnetic field of 0.5 T and magnetic field gradient of 24 T/m. SSE sequence based on stimulated echo, with τ in the range of 0.02–0.2 ms and time between diffusion weighting impulses (∆) of 10 ms was used. For each sample, slices of 200 µm at the same depth of 3500 µm for each sample were examined. Diffusion coefficients were temperature corrected to a mean temperature of 26.1 °C.

An Anton Paar Physica MCR-301 rheometer (Graz, Austria) with parallel plates (PP25) served to the rheometric measurements. Complex viscosity was measured for the hydrogel samples 24 h after polymerization. Disc-shaped samples were casted externally and then loaded between the plates. The samples were subjected to 0.3% strain with 1 Hz by 5 min. The gap was 1 mm width. Measurements were carried out in ambient temperature and reiterated three times for every formula.

The pH of the silicate-acrylate mixtures was measured before the polymerization process, i.e., before introducing the cross-linking monomer and initiators into the system. A Jenway model 3540 pH-meter was used for this purpose.

A Nova Nano SEM 200 scanning electron microscope (Hillsboro, CA, USA) equipped with energy-dispersive X-ray spectroscopy (EDS) analyzer was used for scanning electron microscope (SEM) examination of the hydrogel’s structure and surface. Dried hydrogels were sputtered with carbon with the use of Q150T sputter coater (Laughton, UK) and later observed in high vacuum mode.

## 3. Results and Discussion

### 3.1. TG/DSC Analysis of Polymer-Silicate Hydrogels

**Figure 2:**
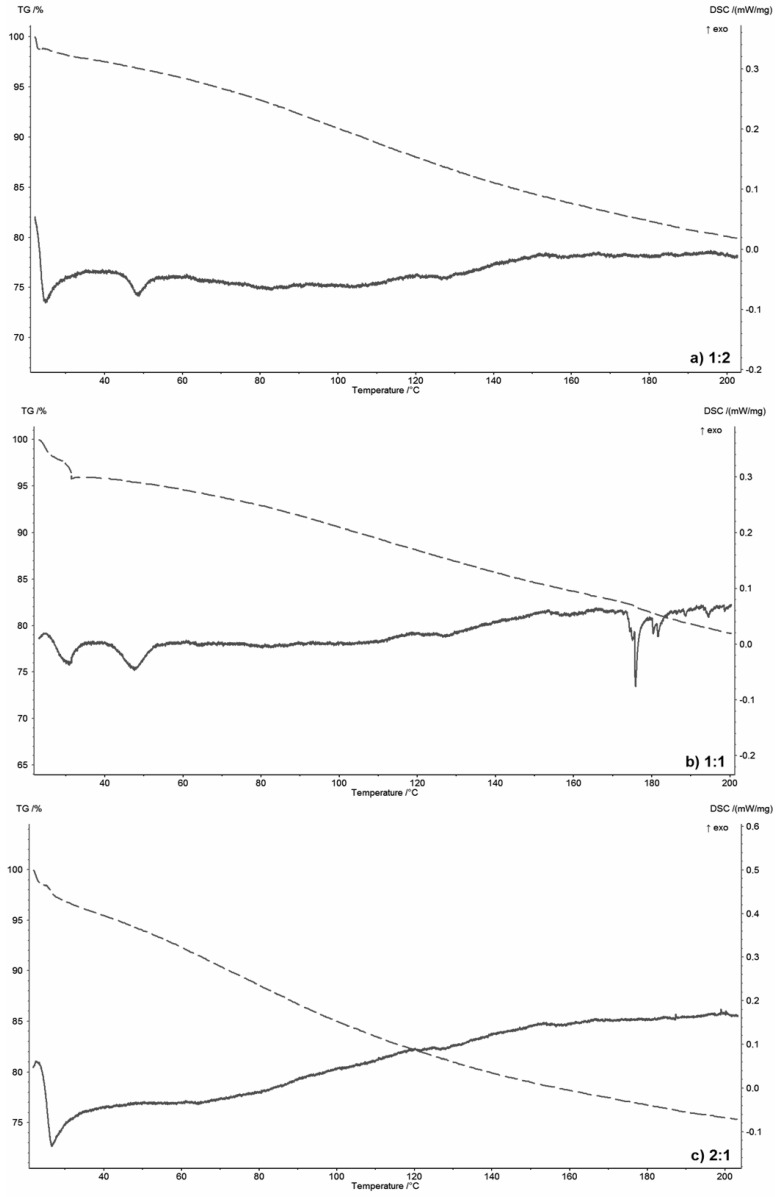
TG/DSC thermograms of the selected polymer-silicate hydrogels with the mass ratio of sodium water glass to polymer: (**a**) 1:2, (**b**) 1:1, (**c**) 2:1.

**Figure 3:**
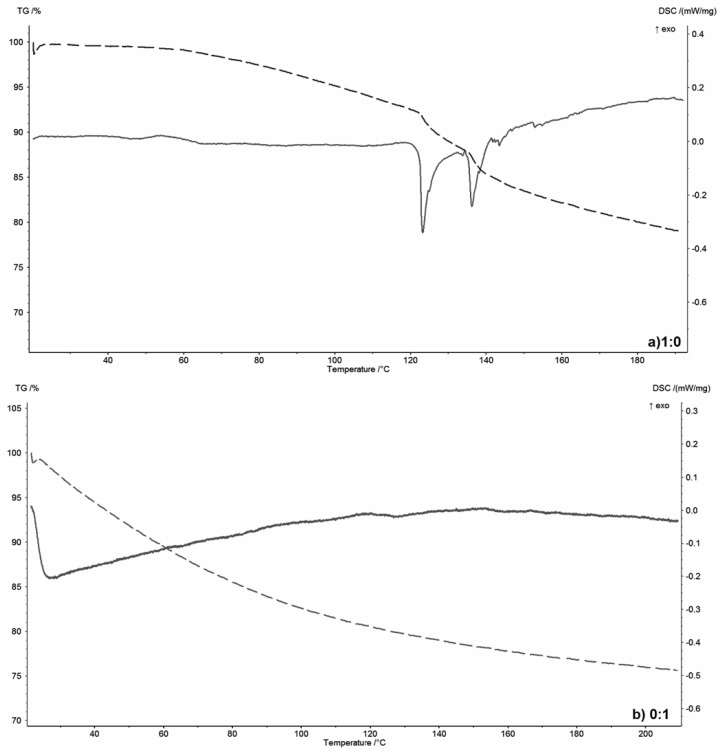
TG/DSC thermograms of the pure components: (**a**) R-150 sodium water glass (1:0), (**b**) sodium polyacrylate (0:1).

Figure 2 shows the thermograms of the selected polymer-silicate hydrogels, while, for comparison, Figure 3 shows the thermograms of pure R-150 water glass (Figure 3a) and pure sodium polyacrylate (Figure 3b). The aim was to consider the behavior of water in these systems; therefore, low operating temperatures of the device up to 200 °C, and the slowest possible heating time (2 °C/min) were used.

Water glass contains about 55 wt.% of water that interacts with silicate structures through intermolecular interactions. Due to the dissociated structure of the water glass, hydrogen and ionic bonds will occur in this system, which is confirmed by the DSC curve. Two endothermic peaks at around 120 °C and 140 °C respectively are visible in Figure 3a. They indicate the presence of at least two types of water–oxygen interactions derived from the silicate domain. Only water can evaporate in this temperature range, since silicate structures can only undergo polymorphic changes. In the case of pure sodium polyacrylate, in the temperature range up to 200 °C, no thermal effects are observed on the DSC curve. Only weight loss due to the water evaporation because of the heat. Water is strongly bound in the network of the polymer structure, and its release does not occur at a temperature of at least up to 200 °C [44,45].

However, analyzing thermograms (Figure 2) of polymer-silicate hydrogels with a mass ratio of 1:2, 1:1 and 2:1 of water glass to polymer, two small endothermic peaks were found in samples 1:2 and 1:1 at 20 °C and 50 °C, while in the 2:1 sample only at 20 °C. These effects are accompanied by about a 10 wt.% loss of weight. These processes are related to the polymerization of acrylates and the beginning of the swelling process. An additional endothermic peak, which is most likely associated with the release of the part of the water associated with silicate structures, was observed at a temperature of about 180 °C on the thermogram of the 1:1 sample (Figure 2b). It could also be associated with the glass-transition temperature (Tg) of the sodium polyacrylate, which for hybrid systems appears at much higher temperatures than for pure polyacrylates (105 °C). However, this effect was not visible on the thermogram of the pure polymer (Figure 3b). Compared to the pure water glass sample (120 °C and 140 °C), this peak is shifted towards higher temperatures, because both silicate structures and water are “retained” in the polymer network, so the energy needed to detach the water molecule is much higher due to the presence of additional intermolecular interactions.

Analyzing the weight loss read from the TG curves, it was found that for dried samples of the pure materials (Figure 3), the weight loss up to 200 °C is about 23 wt.%, while for polymer-silicate hydrogel samples this distribution depends on the mass ratio of the water glass to the polymer and increases with increasing water content in the primary sample.

The biggest weight loss (about 25 wt.%) was noted for the 1:2 sample, i.e., with the highest polymer and water content in the starting material, while the smallest weight loss was noted for the 2:1 sample (about 15 wt.%), i.e., with the lowest polymer and water content and the highest silicate derivatives content derived from water glass.

Based on the above analyses, it can be concluded that the polymer-silicate hydrogel is a mixture of sodium water glass and sodium polyacrylate, where both components do not undergo a chemical reaction, but only interact with each other and with different forms of water by means of the intermolecular bonds, including hydrogen and ionic bonds. Analyzing the TG curves, it can also be concluded that water is less bound in systems with higher water glass content (20 wt.% mass loss for a sample with about 33 wt.% of water glass (sample 1:2) and 25 wt.% mass loss for a sample with about 76 wt.% of water glass).

### 3.2. MIR Analysis of the Polymer-Silicate Hydrogels Structures

**Figure 4:**
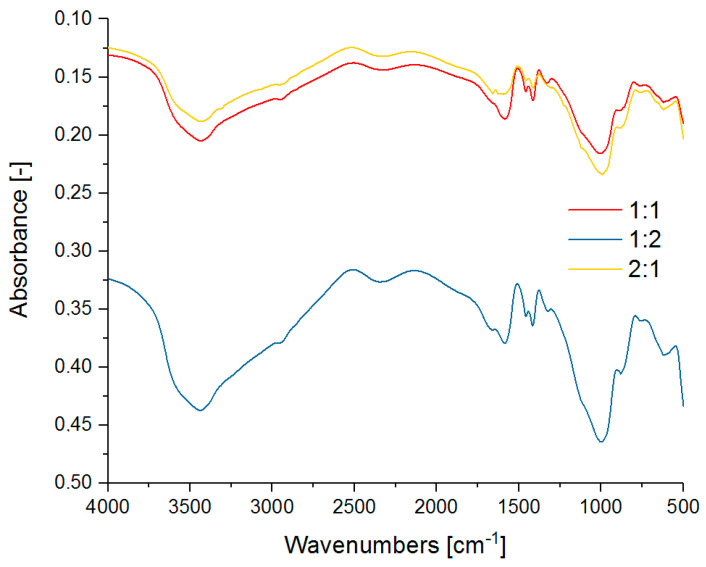
MIR spectra of the selected polymer-silicate hydrogels with the mass ratio 1:1, 1:2 and 2:1 of the sodium water glass to the sodium polyacrylate.

**Figure 5:**
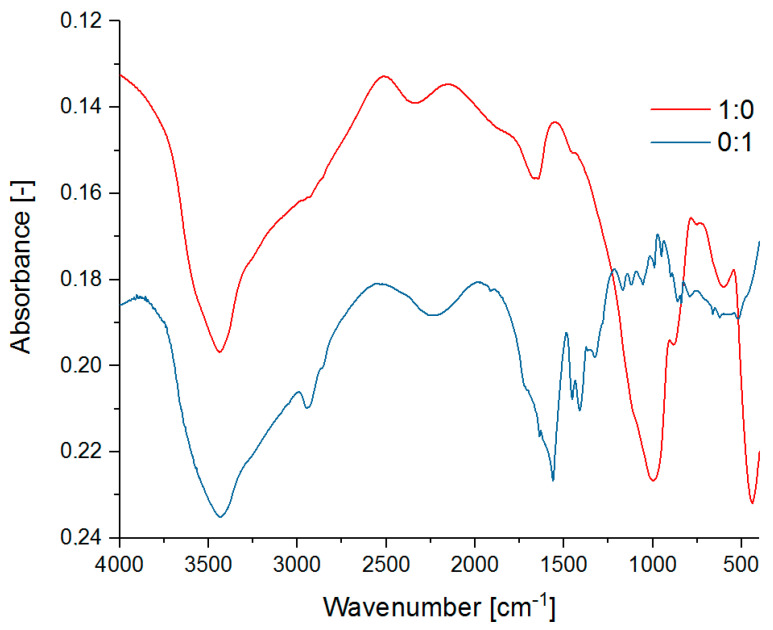
MIR spectra of the pure R-150 sodium water glass (1:0) and the cross-linked sodium polyacrylate (0:1).

The aim of the MIR analysis was to determine the stability of the chemical structure of the polymer-silicate hydrogels (Figure 4). The spectra of pure R-150 sodium water glass and pure cross-linked sodium polyacrylate were also analyzed as the reference samples (Figure 5). It was checked whether a new type of organosilicon structure or the polymer-silicate mixture with two interpenetrating structures that interact only with intermolecular bonds was obtained by hybrid polymerization. We also tried to isolate the bands from the vibrations of different types of water contained in the network of the analyzed hydrogels.

The spectra of pure sodium water glass shows strong absorbance in the 3600–3400 cm^−1^ range due to stretching vibrations of –OH groups of the overlapping water molecules and silanol bonds (Si–OH). The existence of silicate bonds is also evidenced by the bands with a maximum at 1000 cm^−1^, 883 cm^−1^, 611 cm^−1^, and 444 cm^−1^, which originate respectively from: vibrations of SiO_2_ particles, stretching vibrations of Si–O–Si bridges, cyclosilicate vibrations, and bending vibrations of O–Si–O. The absorption band at 1646 cm^−1^ also indicates the presence of both constitutional and molecular water. The spectrum of cross-linked sodium polyacrylate shows the presence of amine bonds derived from NNMBA (2250 cm^−1^ i 785 cm^−1^). A weak band characteristic of stretching vibrations of the –CH and –CH_2_- groups is observed at 2946 cm^−1^. Its shift towards higher wave numbers may indicate the presence of longer aliphatic chains of the cross-linked polyacrylate. There are two bands corresponding to the stretching vibration of the carboxyl group in the wavelength range of 1400–1550 cm^−1^.

No significant band shifts characteristic of the formation of new chemical bonds were observed, and individual ranges of wave numbers can be assigned to the pure substances in the unchanged state. The spectra of the polymer-silicate hydrogel sample with the 1:1 mass ratio is characterized by bands derived from the silicate group bonds at very similar wave number values as for the sample containing only water glass. The bands of the vibration coming from Si–O bonds, that were quite clearly visible in the sample of the pure water glass, might overlap here with the SiO_2_ band, thus giving one broad band with the maximum at 1004cm^−1^. In addition, high absorbance in the range of 1330–1400cm^−1^ and at about 1580 cm^−1^ associated with C–O and C–C bonds respectively, indicates the presence of polyacrylate chains.

In this spectra, due to the –OH stretching vibrations, a wide band at about 3430 cm^−1^ is also observed. In the sample in which the ratio of the sodium water glass to the sodium polyacrylate was 1:2, once again we saw bands derived from the silicate groups, but absorbance values appearing to be slightly lower. The biggest difference in intensity can be seen during the comparison of the bending vibration bands in water. Its shift from 1643 cm^−1^ to 1666 cm^−1^ is also noticeable. This may be due to the binding of more water, which means that less water is present in the free state. Slight shifts relative to the sample with a 1:1 mass ratio were also observed, i.e., for the Si–O–Si vibrations (from 891 to 883 cm^−1^) and vibrations of amino groups (from 2320 to 2347 cm^−1^). The spectrum of the sample with the highest content of the sodium water glass in relation to the sodium polyacrylate, i.e., 2:1, shows very well all bands corresponding to the silicate bonds, which have been identified for the pure sodium water glass.

The stretching band derived from the hydroxyl group at 3460 cm^−1^ and the bending vibration band in the water molecule at 1660 cm^−1^ are also clearly discernible. In contrast, the bands derived from polyacrylate chain bonds are weaker in comparison to the samples with a higher content of this polymer in the composition [46,47,48,49,50,51,52].

Based on the above analyses, it can be concluded that we have obtained a hybrid hydrogel structure where two networks interpenetrate each other. As in the case of TG results, the shift of the characteristic bands of water indicates stronger binding of water in the samples with the higher polymer content.

### 3.3. Mobility of Water in a Polymer-Silicate Hydrogel by Means of ^1^H Low Field NMR

**Figure 6:**
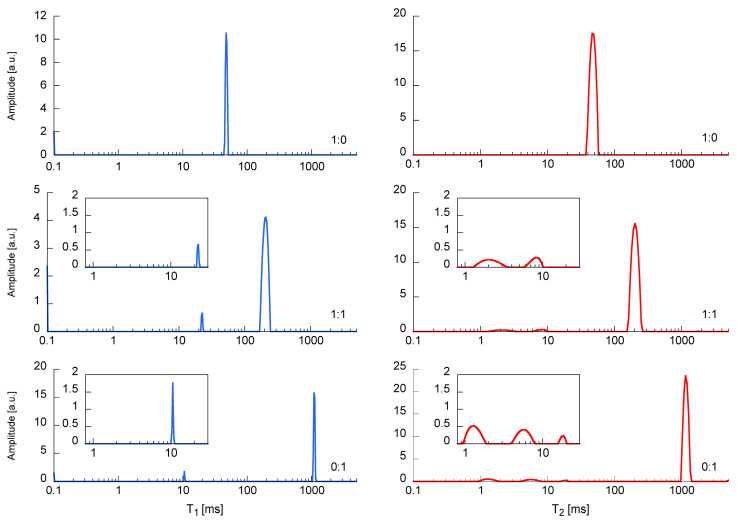
T_1_ (left side) and T_2_ (right side) relaxation times distributions for the selected samples: sodium water glass R-150 (1:0), polymer-silicate hydrogel of the 1:1 mass ratio (1:1), cross-linked sodium polyacrylate (0:1).

**Figure 7:**
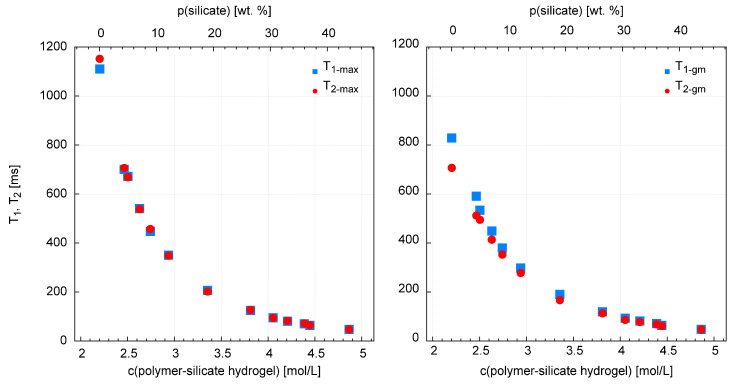
Relaxation times at maximum, T_1 max_, T_2 max_ and for geometric mean, T_1 gm_ and T_2 gm_ for samples with different molar concentrations of polymer-silicate hydrogel. The left most point corresponds to a pure acrylate solution, while the one on the far right corresponds to a sodium water glass solution. From left to right, the acrylate content gradually decreases and the sodium water glass content in the solution increases.

As part of the NMR studies, changes in longitudinal relaxation times—T_1_ and transverse relaxation times—T_2_ were determined. The corresponding alterations in the translational diffusion coefficient D were examined in parallel. Measurements of these parameters were made for different values of molar concentration of the mixture (Figure 6 and Figure 7).

T_1_ spin-lattice relaxation time describes the exchange of proton energy with the entire network. In the case of T_2_ spin-spin relaxation, energy exchange is observed in its immediate vicinity. In both cases, relaxation processes in aqueous solutions contain rotational and translational components regarding the movement of water particles as a result of Brownian motion [53]. Usually, the main sources of relaxation are dipolar interactions (dipolar coupling) between the same type of nuclei (H-H) called homonuclear coupling or different nuclei, e.g., (H-C) referred to as heteronuclear coupling. In polymer solutions, chemical exchange may occur between the structural elements and the solvent. There is a scalar type I effect associated with this, which is a source of an additional relaxation process [54]. In turn, a scalar effect of the second type is a strong relaxation mechanism associated with nuclei having a quadrupole moment. Transversal scalar relaxation of the second type was found for aqueous solutions of paramagnetic ions [54,55]. The relaxation time is then determined by the interaction of water protons and the magnetic moment of ions having unpaired electrons.

Our mixture can be treated as aqueous solutions of two salts: sodium acrylate and sodium silicate. After dissociation, aqueous diamagnetic sodium ions and silica particles (colloidal sols) fill the spaces in the porous acrylate skeleton.

#### 3.3.1. T_1_, T_2_ Relaxation Time Measurements

Due to the lack of an admixture of paramagnetic ions, the relaxation processes will mainly concern homonuclear and heteronuclear dipole interactions of the following proton populations:

– Bulk water;
– Water dipoles bound by Coulomb forces in the hydrated double layer around sodium cations [56];
– Some protons chemically bonded to the silica backbone in the form of silanols (SiOH);
– Water molecules absorbed on the silica surface and surrounding negatively ionized silica particles, forming a hydrated shell [57];
– Hydrogen and carbon atoms forming the acrylate structure (C–H, C–H_2_).

In addition, we can expect scalar effects of the first type regarding chemical exchange and magnetization transfer between the abovementioned different proton pools (cross relaxation) [58].

**Figure 8:**
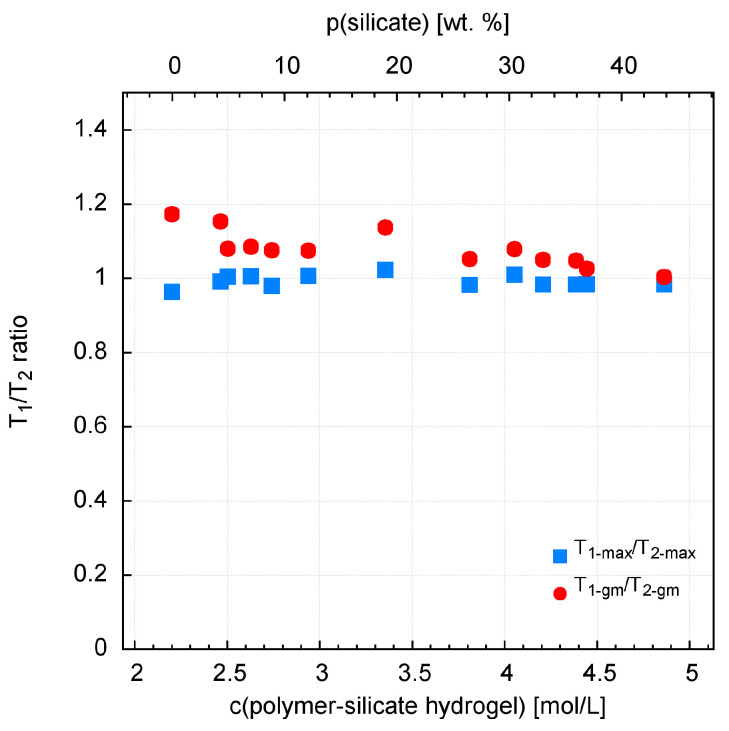
T_1_/T_2_ ratio for the polymer-silicate hydrogel samples.

Observation of the T_1_ and T_2_ distributions and the T_1_/T_2_ ratio leads to the following statements/remarks (Table S1—supplementary material). The longest values of T_1_ and T_2_ are recorded for the highest polymer content in the solution (only/pure acrylate), T_1 max_ = 1110 ms, T_2 max_ = 1152 ms, T_1 gm_ = 829, and T_2 gm_ = 707 ms. The shortest relaxation times are measured for the highest content of water glass in the solution (sample without acrylate) T_1 max_ = 47.1 ms, T_2 max_ = 47.5 ms, T_1 gm_ = 47.9 ms, and T_2 gm_ = 47.3 ms. In addition, there are two clear distributions of relaxation times. The dominant one, which contains more than 90% of the signal and for which the T_1 max_/T_2 max_ ratio is almost perfectly equal to 1 (Figure 6). This proves the ideal averaging of spin relaxation due to the rapid reorientation of water particles (extreme narrowing case) and the rapid exchange of water molecules between relaxation centers. It also confirms the negligible impact of scalar effects [53]. The second distribution, containing a few percent of the signal, with relaxation times to the order of a few milliseconds, is clearly visible with increasing content of acrylate in the mixture. This is accompanied by an increase in the T_1 gm_/T_2 gm_ ratio (Figure 8), which indicates a lack or slow exchange of molecules with the main relaxation centers. This distribution is therefore associated with heteronuclear dipolar coupling between the H–C spins of the acrylate backbone.

#### 3.3.2. D Diffusion Coefficient Measurements

**Figure 9:**
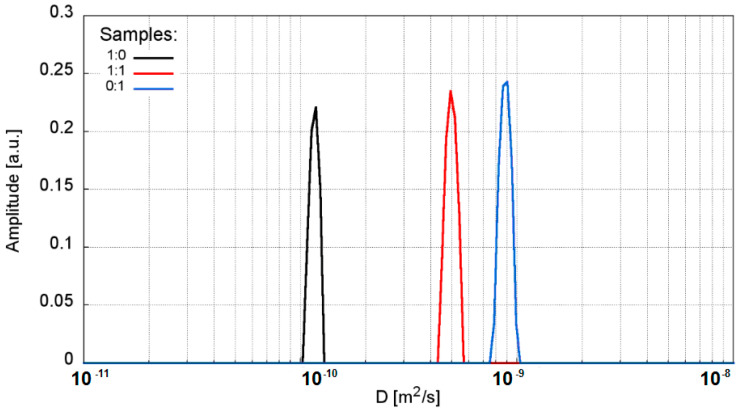
Diffusion coefficient distributions for the selected samples: sodium water glass R-150 (1:0), polymer-silicate hydrogel of the 1:1 mass ratio (1:1), cross-linked sodium polyacrylate (0:1).

**Figure 10:**
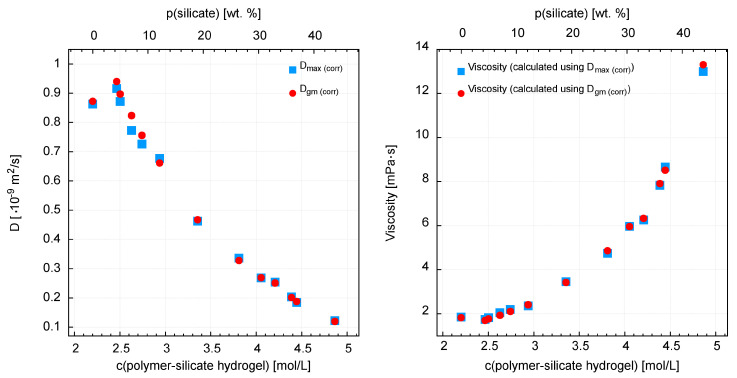
Decrease of diffusion coefficient for the polymer-silicate hydrogels and corresponding viscosity values.

Translational diffusion coefficients significantly decrease from the highest value of D = 0.97 × 10^−9^ m^2^/s for a pure sodium polyacrylate solution, to the lowest value of 0.12, which was recorded for a solution containing only sodium water glass (Figure 9) (Table S2—supplementary material). The variability between these points is slightly exponential (Figure 10). Interestingly, when assessing the samples visually, we can see the highest mobility for the sodium water glass solution, and the lowest for the pure polymer solution. In fact, however, it appears that the water molecules have the least mobility in the sodium water glass solution and the greatest mobility in the pure polymer solution.

Generally, in aqueous silicates, proton (^1^H) populations (except free water) occur in the form of OH hydroxyl groups or strongly bound molecular water [41,59]. The degree of binding of water molecules depends on the structure and quantity of silica, in synthetic [41,60] and natural systems [61]. This is a phenomenological explanation. So as the amount of water glass increases, so does the amount of silica, which plays a key role in limiting the mobility of water molecules. From the point of view of theory, it is as follows. The quotient T_1_/T_2_, which is practically equal to 1, proves ideal averaging of spin relaxation due to rapid reorientation of water particles (extreme narrowing case) and rapid exchange of water molecules between relaxation centers. So, let’s see what it looks like from the T_1_ level, which describes the interaction of spins with the entire network (lattice) [53,54]. Namely, relaxation rate R1 = 1/T_1_ in such a case is proportional to the correlation time τ_c_, and its translational part is approximated by the equation τ_c_ = r2/12D. So we have a linear relationship between T_1_ and D. Thus, the interaction of protons (^1^H) with silica determines the process of limiting the mobility of water in mixtures. The effect of sodium ions is less significant in this respect [56]. 

### 3.4. Viscoelastic Properties of the Polymer-Silicate Hydrogels

**Figure 11:**
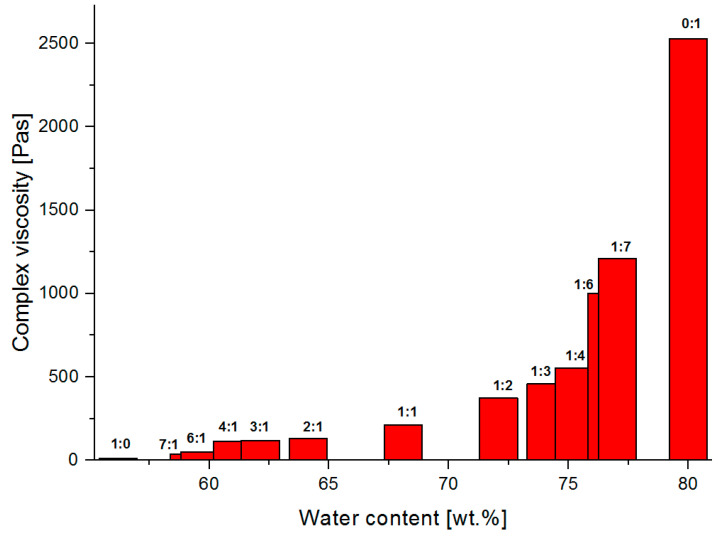
The dependence of the complex viscosity versus water content of the tested polymer-silicate hydrogel samples.

**Figure 12:**
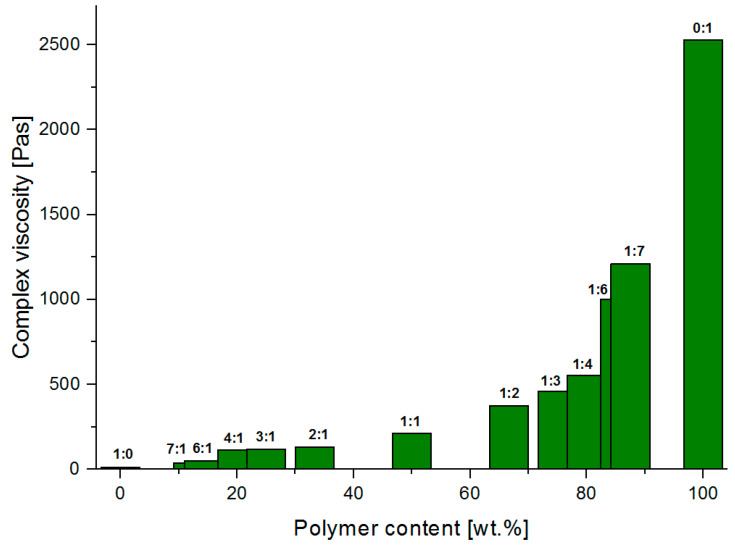
The dependence of the complex viscosity versus polymer content of the tested polymer-silicate hydrogel samples.

**Figure 13:**
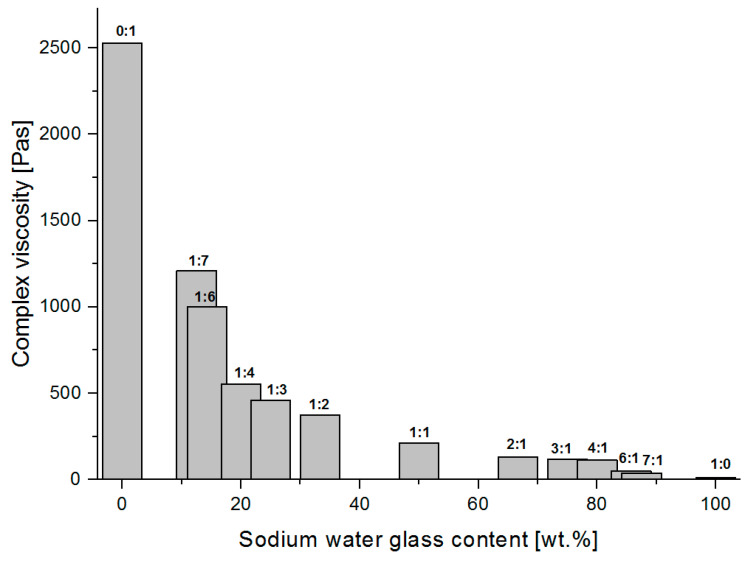
The dependence of the complex viscosity versus sodium water glass content of the tested polymer-silicate hydrogel samples.

Figure 11, Figure 12 and Figure 13 present graphs of the dependence of complex viscosity on the percentage concentration of the selected polymer-silicate hydrogel components, respectively the percentage concentration of the water (derived from both sodium water glass and polymer), the percentage concentration of the polymer (derived from the initial percentage of aqueous sodium acrylate), and the percentage concentration of the sodium water glass (the initial percentage of the R-150 sodium water glass).

Analyzing the above graphs, it can be seen that the highest complex viscosity is to be found with pure cross-linked sodium polyacrylate, where the water content in the initial sample was 80 wt.%. Samples with the mass ratio of the water glass to the polymer from 1:1 to 1:7, where the viscosity ranges from 211–1210 Pas, were solid-like after polymerization, and their complex viscosity increased along with the polymer content in the composition. Samples with the mass ratio of water glass to the polymer from 2:1 to 7: 1, where the viscosity was in the range from 129–139 Pas, were liquid-like after polymerization. This makes it possible to conclude that the cross-linked sodium polyacrylate is a hybrid hydrogel matrix, in the pores of which are water molecules and silicate structures that interact with each other through the intermolecular interactions (based also on the interpretation from TG/DSC and MIR results), i.e., hydrogen bonds and ionic bonds. The strongly integrated structure of the analyzed hybrid hydrogel is weakened by the increasing content of the water glass, which seems to be obvious, as the silicate structures compete for space with the water molecules. According to the literature data, their sizes can reach up to 700 nm, i.e., they are much larger than a single molecule of water (2.75 A). They occupy the largest spaces, and the rest is filled with water molecules that can merge into larger clusters, such as aggregates, etc., that are still much smaller than the silicate structures. The structure of the polymer network is also weakened by the high pH that characterizes the water glass. The pH of the mixtures measured before polymerization ranged from 12.65 (1:1 mass ratio of the water glass to the polymer) to 12.74 (7:1 mass ratio of the water glass to the polymer) (Table 2). A strongly alkaline environment significantly accelerated the polymerization process (from about 20 min to even 5 min). Acrylic monomers are very sensitive to pH changes. The degree of polymerization already accelerates significantly at above pH = 7. Its maximum is pH = 10, and then drops again in the range of pH 11–12. This phenomenon is explained by the reduction in the degree of polymer chain termination caused by the repulsion of polyions with the same charge [52,62,63,64,65,66,67,68,69,70].

### 3.5. SEM Analysis of the Polymer-Silicate Hydrogels

**Figure 14:**
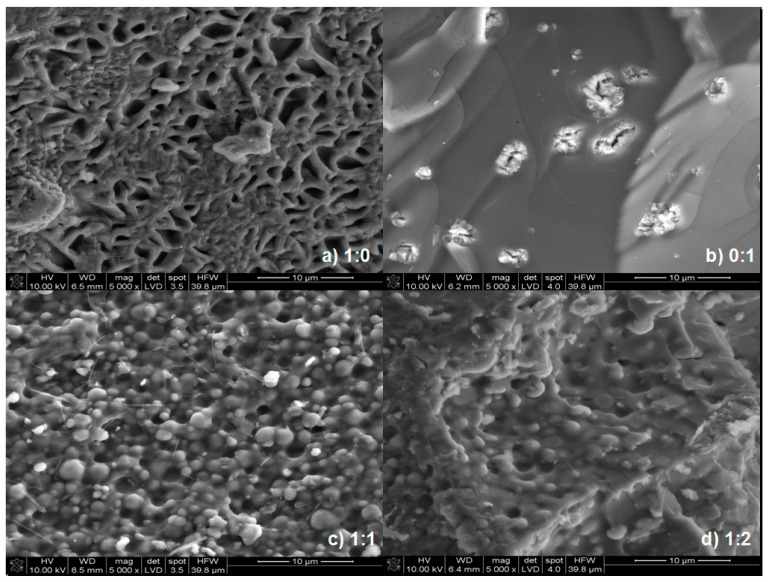
SEM microphotographs: (**a**) R-150 sodium water glass (1:0), (**b**) cross-linked sodium polyacrylate (0:1), (**c**) hydrogel sample of the 1:1 mass ratio of the sodium water glass to the sodium polyacrylate, (**d**) hydrogel sample of the 1:2 mass ratio of the sodium water glass to the sodium polyacrylate.

Figure 14 contains SEM micrographs of the dried samples of the polymer-silicate hydrogels (samples 1:1 and 1:2) and, for comparison, dried samples of R-150 sodium water glass and cross-linked sodium polyacrylate.

**Figure 15:**
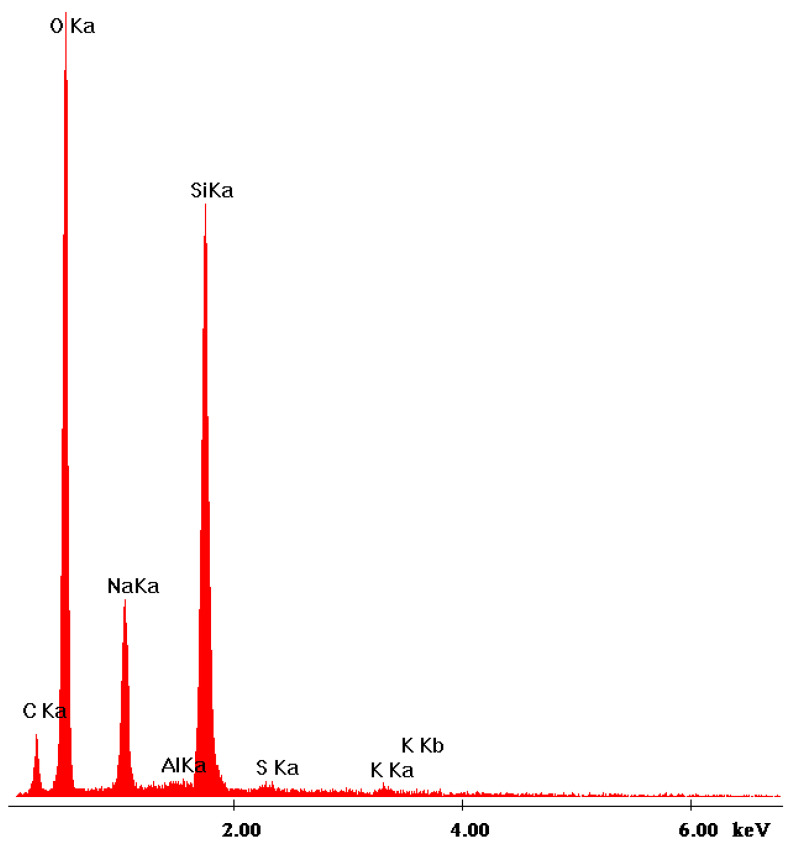
EDS analysis of the 1:1 mass ratio of the sodium water glass to the sodium polyacrylate.

The microphotographs show significant differences between pure sodium polyacrylate, sodium water glass and mixtures thereof. Water glass creates a skeletal structure, with a clear texture and elongated shape of crystallites. However, when there was more water glass in the sample, the presence of lighter oval-shaped fields, which are partially precipitated silica (Figure 15), was found. This may suggest that the gelation of the examined systems not only occurs by polymerization, but also by the precipitation of silicate forms under the influence of changing pH.

As the cross-linked sodium polyacrylate is added, the crystallites become rounded, the internal structure is blurred, and the crystallites are less and less visible. A completely smooth surface that creates a strongly integrated monolithic structure, cut with irregular gaps, which are probably a cross-linking defect, is observed in the case of pure sodium polyacrylate. Based on the microphotographs of the hydrogel samples after drying, it was difficult to determine the relationship between the amount of water glass or polymer. It was only noted that the more polymer that was in the sample, the smoother the surface of the sample was. Open porosity was not found in the analyzed microphotographs. Based on Figure 14c,d, it can be assumed that there is so-called closed porosity.

## 4. Conclusions

The research and analyses of polymer-silicate hydrogels allowed for the following conclusions to be drawn:

– Polymerization of the mixture of sodium water glass and sodium acrylate in the presence of the NTS/KPS redox initiator system and NNMBA cross-linking monomer results in the formation of a hybrid structure with a porous polyacrylate scaffolding filled with a solution of silicate derivatives;
– In the resulting hybrid structure, water is more strongly bonded through intermolecular interactions with both the polymer scaffolding and silicate structures, and because of that can evaporate at higher temperatures, even at 180 °C;
– Although the alkaline environment accelerates the polymerization process, it also weakens the structure of the resulting hydrogel with the increased content of water glass. It can be explained by the reduction in the degree of polymer chain termination caused by the repulsion of polyions with the same charge;
– The mobility of water molecules decreases as the amount of water glass in the mixture increases, which contradicts visual observation;
– Averaging the value of relaxation times and diffusion coefficients indicates a rapid exchange (diffusional coupling) between both the pores of the acrylate as well as the hydration layers around sodium ions and silica particles. The exception is the signal recorded due to the heteronuclear dipolar couplings of C–H dipoles contained in the acrylate backbone, which indicates the absence or very slow exchange with other proton centers;
– It is possible to synthesize stable silicate-polymer hydrogels at high pH, where, by adding the sodium water glass, the water capacity of the hydrogel can be controlled, as well as relaxation time what is useful when designing medical phantoms.

## Figures and Tables

**Table 1:** Composition of the tested polymer-silicate hydrogels.

Sample Number	Mass Ratio of WG to pANa	WG [wt.%]	pANa [wt.%]	Ana [wt.%]	SiO_2_ [wt.%]	Na_2_O [wt.%]	H_2_O [wt.%]	H_2_O_(p)_ * [wt.%]	H_2_O_(wg)_ ** [wt.%]	Sample Form
1	SW R-150	100.00	-	-	29.10	14.70	56.20	-	56.20	liquid-like
2	pANa	-	100.00	20.00	-	-	80.00	80.00	-	solid-like
3	1:1	50.00	50.00	10.00	14.53	7.37	68.10	40.00	28.10	solid-like
4	1:2	33.33	66.67	13.33	9.70	4.90	72.07	53.33	18.73	solid-like
5	1:3	25.00	75.00	15.00	7.27	3.67	74.07	60.00	14.07	solid-like
6	1:4	20.00	80.00	16.00	5.83	2.93	75.23	64.00	11.23	solid-like
7	1:6	14.30	85.70	17.13	4.17	2.10	76.60	68.57	8.03	solid-like
8	1:7	12.50	87.50	17.50	3.63	1.83	77.03	30.00	7.03	solid-like
9	2:1	66.67	33.33	6.67	19.40	9.80	64.13	26.67	37.47	liquid-like
10	3:1	75.00	25.00	5.00	21.83	11.03	62.13	20.00	42.13	liquid-like
11	4:1	80.00	20.00	4.00	23.27	11.77	60.97	16.00	44.97	liquid-like
12	6:1	85.70	14.30	2.87	24.93	12.60	59.60	11.43	48.17	liquid-like
13	7:1	87.50	12.50	2.50	25.47	12.87	59.17	10.00	49.17	liquid-like

* water derived from polymer ** water derived from sodium water glass.

**Table 2:** pH of the acrylate-silicate mixtures before the polymerization.

Sample Symbol	1	2	3	4	5	6	7	8	9	10	11	12	13
pH	13.45	7.1	12.65	12.49	12.35	12.31	12.24	12.17	12.65	12.61	12.71	12.73	12.74

## Supplementary Materials

The following are available online at https://www.mdpi.com/1996-1944/13/18/4092/s1, Table S1: T_1_ and T_2_ relaxation times: values for maximal amplitude and geometric mean (log-mean), Table S2: Diffusion coefficients of water molecules and viscosities of the samples calculated on the base of diffusion results.
