# Supplementary material for: Water Interactions in Hybrid Polyacrylate-Silicate Hydrogel Systems
Source: Materials (Basel). 2020 Sep 15;13(18):4092. doi: 10.3390/ma13184092 (PMC7560421; doi:10.3390/ma13184092)
Supplement: Supplementary file 1 [file materials-13-04092-s001.pdf]

T1, T2 relaxation time measurements

Table S1. T_1 and T_2 relaxation times: values for maximal amplitude and geometric mean (log-mean).

Sample number	Mass ratio (SW:pANa)	SW [wt. %]	pANa [wt. %]	H_2O [wt. %]	cCSW ($Na_2O \cdot SiO_2$) [mol/L]	c(ANa) [mol/L]	$T_{1\ max}$ [ms]	$T_{2\ max}$ [ms]	$T_{1\ gm}$ [ms]	$T_{2\ gm}$ [ms]	$T_{1\ max}/T_{2\ max}$	$T_{1\ gm}/T_{2\ gm}$
1	0:1 (pANa)	0.0	20.0	80.0	0.00	2.60	1100	1152	829	707	0.964	1.173
2	1:7	5.5	17.5	77.0	0.36	2.25	700	706	591	512	0.991	1.154
3	1:6	6.3	17.1	76.6	0.41	2.22	672	669	534	495	1.005	1.079
4	1:4	8.8	16.0	75.2	0.59	2.10	541	538	449	414	1.006	1.085
5	1:3	10.9	15.0	74.1	0.74	1.99	448	457	379	353	0.979	1.075
6	1:2	14.6	13.3	72.1	1.01	1.81	351	348	299	278	1.007	1.074
7	1:1	21.9	10.0	68.1	1.58	1.42	207	202	190	167	1.022	1.137
8	2:1	29.2	6.7	64.1	2.21	0.99	125	127	119	113	0.982	1.052
9	3:1	32.7	5.0	62.0	2.54	0.76	95.4	94.5	93.5	86.7	1.010	1.079
10	4:1	35.0	4.0	61.0	2.75	0.62	81.1	82.5	81.6	77.7	0.983	1.050
11	6:1	37.5	2.9	59.6	3.00	0.45	70.8	72.0	71.5	68.3	0.984	1.047
12	7:1	38.3	2.5	59.2	3.07	0.39	63.5	64.6	63.6	61.9	0.984	1.026
13	1:0 (SW)	43.8	0.0	56.2	3.51	0.00	47.1	47.5	47.5	47.3	0.984	1.004

D diffusion coefficient measurements

Table S2. Diffusion coefficients of water molecules and viscosities of the samples calculated on the base of diffusion results.

Sample number	Mass ratio (SW:pANa)	$D_{max(temperature\ corrected)}$ [*10^{-9} m^2/s]	$D_{gm(temperature\ corrected)}$ [*10^{-9} m^2/s]	η (calculated using $D_{max\ (corr)}$)	η (calculated using $D_{gm\ (corr)}$)
1	0:1 (pANa)	0.86	0.87	1.85	1.83
2	1:7	0.92	0.94	1.74	1.70
3	1:6	0.87	0.90	1.83	1.77
4	1:4	0.77	0.82	2.06	1.93
5	1:3	0.73	0.76	2.20	2.11
6	1:2	0.68	0.66	2.36	2.41
7	1:1	0.46	0.47	3.46	3.43
8	2:1	0.34	0.33	4.75	4.87
9	3:1	0.27	0.27	5.97	5.96
10	4:1	0.25	0.25	6.26	6.33
11	6:1	0.20	0.20	7.84	7.91
12	7:1	0.18	0.19	8.66	8.52
13	1:0 (SW)	0.12	0.12	13.0	13.3
